# Huddersfield Infirmary

**Published:** 1893-02-18

**Authors:** 


### The Institutional Workshop.

### WITHIN THE HOSPITALS.

### HUDDERSFIELD INFIRMARY.

(By Our Special Commissioner.)

This institution occupies a conspicuous site in the main thoroughfare from the railway. It has been erected in the public gardens, and connected with it are baths, which are also open to the public. These grounds are very tastefully laid out, and although the atmosphere is against floriculture, still the results are strikingly picturesque, and on the whole satisfactory. The infirmary contains upwards of one hundred beds, and its invested funds amount to £40,000. In addition to this, there is a special building fund, invested chiefly in colonial stocks, which approaches £10,000. The infirmary relies more upon the annual subscriptions than the interest upon its invested funds, the former amounting to about £1,700, and the latter to £1,650. The workpeople of Huddersfield contribute about £1,000 a year; the Hospital Saturday Fund realises about £800 more. The income last year approached £6,000, and the expenditure was within £5,800. It appears that the sale of trusses, bandages, and surgical instruments supplied to the patients amounted to upwards of £64 last year. We do not remember to have seen any similar item in the accounts of any other hospital.

Buildings.

The hospital proper consists of the administrative section, at the end of which are four wards and two wings, one of which is of two storeys, and contains two large wards each with 32 beds. The other is a much smaller and narrower ward of early construction, which will presumably be pulled down when the new buildings are erected. The appearance of these wards is very satisfactory, and bears evidence of careful administration and close attention to detail. The workpeople's subscriptions show that the hospital is popular, no doubt deservedly so, among the poor of Huddersfield, and the general appearance of the institution led us to conclude that much good work is done within its walls. There were 869 in-patients, 398 out-patients, and 87 home patients, in addition to 1,723 casualties under treatment during the year ending June 30th, 1892. The average number of beds occupied is 81. Each in-patient remains in the hospital for five weeks on an average.

